# Chronic Kidney Disease Risk in African and Caribbean Populations With HIV

**DOI:** 10.1093/infdis/jiy397

**Published:** 2018-07-06

**Authors:** Sophie Jose, Lisa Hamzah, Rachael Jones, Debbie Williams, Alan Winston, Fiona Burns, Andrew N Phillips, Caroline A Sabin, Frank A Post, UK CHIC Study, Jonathan Ainsworth, Sris Allan, Jane Anderson, Abdel Babiker, David Chadwick, Duncan Churchill, Valerie Delpech, David Dunn, Brian Gazzard, Richard Gilson, Mark Gompels, Phillip Hay, Teresa Hill, Margaret Johnson, Sophie Jose, Stephen Kegg, Clifford Leen, Fabiola Martin, Dushyant Mital, Mark Nelson, Chloe Orkin, Adrian Palfreeman, Andrew Phillips, Deenan Pillay, Frank Post, Jillian Pritchard, Caroline Sabin, Achim Schwenk, Anjum Tariq, Roy Trevelion, Andy Ustianowski, John Walsh

**Affiliations:** 1 Institute for Global Health, University College London; 2 Department of Sexual Health, King’s College Hospital NHS Foundation Trust, London; 3 Department of Sexual Health, Chelsea and Westminster Hospital NHS Foundation Trust, London; 4 Department of Sexual Health, Brighton and Sussex University Hospitals NHS Trust; 5 Division of Infectious Diseases, Department of Medicine, Imperial College London, United Kingdom

**Keywords:** Africa, chronic kidney disease, HIV, Caribbean, incidence

## Abstract

We conducted an observational cohort study of end-stage kidney disease (ESKD) in >7000 African and Caribbean people with HIV in the UK. Using Poisson regression and East Africans as the reference group, the adjusted incidence rate ratio (95% confidence interval) of ESKD was 3.14 (1.26–7.84) in Southern Africans, 6.35 (2.53–15.96) in West Africans, and 5.26 (1.91–14.43) in Caribbeans. Higher CD4 cell count and suppressed HIV replication were associated with reduced risk of ESKD. The risk of ESKD varied among HIV-positive people of African heritage, with the highest rates observed in those of West African descent.

## Teaser

People of West-African heritage were noted to be at 6-fold greater risk of end-stage kidney disease compared to those originating from East Africa, which suggests that genetic factors are likely determinants of kidney disease in these populations.

Chronic kidney disease (CKD) is an important and increasing cause of morbidity and mortality in Africa. In black people with human immunodeficiency virus (HIV), HIV-associated nephropathy (HIVAN) is the most severe form of kidney disease [1–3] and a leading cause of end-stage kidney disease (ESKD) [4, 5]. HIV replication and immunodeficiency are potent risk factors for HIVAN, and antiretroviral therapy (ART) reduces the incidence of HIVAN and the risk of kidney disease progression in those with established disease [1–3, 6]. Homozygosity, or compound heterozygosity, for 2 polymorphisms in the apolipoprotein L1 (*APOL1*) gene is strongly associated with HIVAN (odds ratio 29–89) [7, 8]. The distribution of the *APOL1* coding variants varies greatly among sub-Saharan African populations, with the highest frequencies reported in West Africa, lower frequencies in Southern and Central Africa, and the lowest frequency (or, in some populations, absence) in East Africa [9].

A large number of people from different parts of sub-Saharan Africa are resident in the United Kingdom and accessing HIV care, which includes the provision of ART with regular monitoring of kidney function and renal replacement therapy for those with ESKD, thus providing an opportunity within the UK health care setting to study the prevalence and incidence of CKD in African populations. We hypothesized that, in line with the distribution of *APOL1* risk alleles, the rates of severe CKD would be highest among individuals from West Africa, intermediate among those from Southern Africa, and lowest among those from East Africa. For comparison, we also analyzed data from black Caribbean persons (who are predominantly of West African descent).

## METHODS

Data were obtained from the UK Collaborative HIV Cohort (CHIC) Study, an observational cohort study of HIV-positive individuals, aged 16 years and older, who have attended some of the largest HIV clinics in the United Kingdom at least once since January 1996 [10]. The UK CHIC study is approved by the National Health Service Multi-Centre Research Ethics Committee; individual participant consent was not required. The present analyses include data up to December 2014 and were restricted to 15 centers that routinely contributed serum creatinine data.

Country of birth and ethnicity are routinely reported by HIV clinics in the UK. HIV-positive people of black African or “other” black ethnicity with country of birth belonging to sub-Saharan Africa were eligible for inclusion if they had at least 2 serum creatinine measures at least 3 months apart, as were individuals of black Caribbean ethnicity (regardless of country of origin). Four groups of birth/ethnicity categories were created: those from sub-Saharan Africa were grouped into a priori defined regions (East, Southern, or West Africa) based on country of birth (Supplementary Table 1); a fourth category was created for those of black Caribbean ethnicity. Baseline characteristics of the 4 groups were described and compared using *Χ*^2^, Kruskal-Wallis tests, or ANOVA, as appropriate. Serum creatinine measurements were converted to estimated glomerular filtration rate (eGFR) using the CKD-EPI formula [10]; CKD (stage ≥3) and ESKD (stage 5) were defined by at least 2 eGFR <60 and <15 mL/min/1.73m^2^ over >3 months, respectively.

Follow-up was calculated from an individual’s first serum creatinine measurement after the center-specific commencement of routine monitoring of renal function to the earliest of: last UK CHIC follow-up date; death; December 2014; or first occurrence of CKD/ESKD. Kaplan-Meier methods were used to estimate the probability of CKD in the 4 groups. Crude incidence rates (95% confidence interval) were calculated, and Poisson regression was used to assess the association between region of birth/ethnicity and CKD with adjustments for baseline (sex, mode of acquisition) and time-updated factors (age, current and nadir CD4 cell count, acquired immunodeficiency syndrome, ART experience and viral load status, and exposure to tenofovir disoproxil fumarate [TDF] and protease inhibitors [PI]). Age and sex were adjusted for in all models, with other factors included if significant or if they significantly improved model fit. We performed additional analyses in which viral subtype (where available) was included in the multivariable model.

## RESULTS

Of the 19710 individuals of black ethnicity in UK CHIC, 14114 had data on country of birth or self-identified as Caribbean. We excluded 6326 individuals who had no or insufficient creatinine data (mainly people who attended a site that does not supply creatinine data to UK CHIC). This left 7788 participants for analysis: 2033 (26.1%) from East Africa, 3101 (39.8%) from Southern Africa, 1487 (19.1%) from West Africa, and 1167 (15.0%) Caribbeans (Supplementary Figure 1 and Table 1). The median (interquartile range) total number of creatinine measurements was 18 (interquartile range [IQR] 8–37), 14 (IQR 7–27), 14 (IQR 7–28), and 16 (IQR 7–32), respectively, for participants in the 4 groups. The characteristics of those included differed by region/ethnicity, as shown in Table 1. The participants from sub-Saharan Africa had a mean age of 36 years and were predominantly female. Many were ART experienced, with a median CD4 cell count of about 300 cells/mm^3^. The prevalence of hepatitis B was highest among West Africans (10.5%), and few participants were coinfected with hepatitis C. The predominant subtype of HIV was A in East Africa and C in Southern Africa, while circulating recombinant forms (CRF, esp. CRF02_AG) were highly prevalent in West Africa. The Caribbean participants were of similar age to the Africans but more likely to be male, to have acquired HIV through sex between men, and to be infected with HIV subtype B.

**Table 1.** Baseline Characteristics and Incidence of Chronic Kidney Disease During Follow-up, According to Country of Origin

Region	East Africa	Southern Africa	West Africa	Caribbean	*P* Value^a^	*P* Value^b^
N	2033	3101	1487	1167		
Baseline characteristics						
Age						
Mean (SD)	36 (9.0)	36 (9.0)	36 (9.8)	37 (11.7)	.43	.28
Sex, No. (%)						
Male	717 (35.3)	986 (31.8)	618 (41.6)	787 (67.4)	<.001	<.001
Female	1316 (64.7)	2115 (68.2)	869 (58.4)	380 (32.6)		
Mode of acquisition, No. (%)						
Heterosexual	1838 (90.4)	2822 (91.0)	1324 (89.0)	618 (53.0)	<.001	<.001
MSM	54 (2.7)	54 (1.7)	104 (7.1)	498 (42.7)		
Other/unknown	141 (7.0)	224 (7.2)	60 (4.0)	50 (4.3)		
HBV, No. (%) (n = 3797)						
No	894 (95.0)	1322 (93.1)	789 (89.5)	530 (95.7)	<.001	<.001
Yes	47 (5.0)	98 (6.9)	93 (10.5)	24 (4.3)		
HCV, No. (%) (n = 4028)						
No	923 (98.5)	1493 (98.4)	909 (99.0)	641 (97.9)	.39	.060
Yes	14 (1.5)	25 (1.6)	9 (1.0)	14 (2.1)		
HIV subtype, No. (%) (n = 4660)						
A	465 (39.4)	80 (4.6)	56 (5.7)	33 (4.3)	<.001	<.001
B	38 (3.2)	35 (2.0)	72 (7.3)	536 (70.0)		
C	345 (29.2)	1388 (80.3)	74 (7.5)	79 (10.3)		
Any CRF	102 (8.6)	115 (6.7)	616 (62.6)	84 (11.0)		
Other	231 (19.6)	111 (6.4)	166 (16.9)	34 (4.4)		
Prior AIDS, No. (%)						
Yes	417 (20.5)	591 (19.1)	180 (12.1)	151 (12.9)	<.001	.45
ART experienced, No. (%)						
Yes	1076 (52.9)	1595 (51.4)	565 (38.0)	366 (31.4)	<.001	<.001
CD4 count						
Median (IQR)	310 (164–480)	320 (171–490)	298 (148–460)	370 (206–558)	.003	<.001
Viral load						
Median (IQR)	2.9 (1.7–4.4)	3.0 (1.7–4.4)	3.8 (1.8–4.8)	3.9 (2.3–4.7)	<.001	.35
eGFR, No. (%)						
<60 ^c^	21 (1.0)	71 (2.3)	64 (4.3)	30 (2.6)	<.001	.040
60–74	70 (3.5)	97 (3.1)	67 (4.5)	58 (5.0)		
75–89	206 (10.2)	338 (10.9)	173 (11.7)	161 (13.9)		
≥90	1732 (85.4)	2591 (83.7)	1172 (79.4)	913 (78.6)		
Median, IQR	119 (100, 134)	115 (98, 130)	112 (94, 131)	108 (92, 125)	<.001	<.001
Incidence of CKD per 1000 person-years						
CKD, eGFR <60 cutoff						
Follow-up, person-years	15216	18406	8057	7920		
n	41	77	64	49		
%	2.0	2.5	4.3	4.2		
Incidence (95% CI)	2.7 (1.9–3.5)	4.2 (3.3–5.1)	7.9 (6.0–9.9)	6.2 (4.5–7.9)	<.001	.18
ESKD, eGFR <15 cutoff						
Follow-up, person-years	15442	18683	8268	8081		
n	7	22	23	13		
%	0.3	0.7	1.6	1.1		
Incidence (95% CI)	0.5 (0.2–0.9)	1.2 (0.7–1.7)	2.8 (1.6–3.9)	1.6 (0.7–2.5)	<.001	.11

Baseline characteristics were compared using *Χ*^2^, Kruskal-Wallis tests, or ANOVA, as appropriate. Crude incidence rates were compared using Poisson regression.

Abbreviations: AIDS, acquired immunodeficiency syndrome; ART, antiretroviral therapy; CI, confidence interval; CKD, chronic kidney disease; CRF, circulating recombinant forms; eGFR, estimated glomerular filtration rate; ESKD, end-stage kidney disease; HBV, hepatitis B (surface antigen positive); HCV, hepatitis C (antibody positive); HIV, human immunodeficiency virus; IQR, interquartile range; MSM, men who have sex with men.

^a^ *P* value comparison East/Southern/West Africa.

^b^ *P* value West African vs Caribbean.

^c^ CKD ≥3 at baseline in 24 and 3 had ESKD at baseline.

There were significant differences in baseline eGFR among those from different parts of sub-Saharan Africa, with participants from West Africa being less likely to have normal kidney function (eGFR ≥90 mL/min/1.73m^2^) and, correspondingly, more likely to have impaired kidney function (eGFR <60 mL/min/1.73m^2^). Caribbeans had lower median eGFR compared to West Africans although fewer individuals had renal impairment (Table 1). In total, 255 (3.3%) individuals presented with or developed CKD (stage ≥3), and 68 (0.9%) ESKD. The cumulative probability of CKD among participants from sub-Saharan Africa was highest among West Africans, with approximately 6% developing CKD and 2% ESKD by 10–15 years from cohort entry (Supplementary Figure 2). Among individuals without CKD at baseline (n = 7764) and over 49599, and 50474 person-years of follow-up, the overall crude incidence rates for CKD stages ≥3 and 5 were 4.7 (IQR 4.1–5.3) and 1.3 (IQR 1.0–1.6) per 1000 person-years, respectively. Among people from sub-Saharan Africa, West Africans had the highest rates, Southern Africans intermediate rates, and East Africans the lowest rates of CKD. The rates of CKD among Caribbeans were similar, albeit somewhat lower, to those observed for West Africans (Table 1).

In unadjusted analyses with East Africans as the reference group, rate ratio estimates for CKD and ESKD were 2.95 and 6.14 for West Africans, and 1.55 and 2.60 for Southern Africans, respectively. Adjusting for age, sex, current and nadir CD4 cell count, ART, and viral load status had minimal impact on these estimates (Table 2). Higher CD4 cell count, suppressed HIV replication, and exposure to TDF were associated with reduced risk of CKD and ESKD, female sex with reduced risk of ESKD, and older age and PI use with increased risk of CKD. HIV subtype was available for 4660 participants, and specific subtypes (C, CRF, and other) were associated with increased risk of CKD. Adjustment for HIV subtype attenuated the effect of geographic region for CKD but had minimal effect on the association between region and ESKD (Supplementary Table 2).

**Table 2.** Associations Between Region of Birth/Ethnicity Group and CKD/ESKD

	CKD				ESKD			
	Univariable Estimates^a^		Multivariable Estimates^a^		Univariable Estimates^a^		Multivariable Estimates^a^	
	IRR (95% CI)	*P* Value	IRR (95% CI)	*P* Value	IRR (95% CI)	*P* Value	IRR (95% CI)	*P* Value
Region								
East Africa	1		1		1		1	
Southern Africa	1.55 (1.06–2.27)	.0229	1.51 (1.02–2.25)	.0409	2.60 (1.11–6.08)	.0278	3.14 (1.26–7.84)	.0142
West Africa	2.95 (1.99–4.36)	<.0001	2.59 (1.71–3.94)	<.0001	6.14 (2.63–14.3)	<.0001	6.35 (2.53–15.96)	<.0001
Caribbean	2.30 (1.52–3.48)	<.0001	2.06 (1.29–3.31)	.0027	3.55 (1.42–8.89)	.0069	5.26 (1.91–14.43)	.0013
Age^b^								
Per 10 years	1.83 (1.62–2.07)	<.0001	2.08 (1.83–2.36)	<.0001	1.11 (0.86–1.43)	.4395	1.20 (0.91–1.57)	0.1917
Sex								
Male	1		1		1		1	
Female	0.74 (0.57–0.96)	.0238	1.05 (0.78–1.42)	.7336	0.57 (0.35–0.93)	.024	0.54 (0.31–0.93)	.0275
Mode of HIV acquisition								
Heterosexual	1		1		1		1	
Other	0.54 (0.34–0.86)	.0088	0.50 (0.28–0.87)	.0145	0.38 (0.14–1.04)	.0596	0.19 (0.05–0.65)	.0083
Current CD4 count^b^								
Per 50 cells/mm	0.86 (0.84–0.89)	<.0001	0.91 (0.88–0.94)	<.0001	0.85 (0.80–0.90)	<.0001	0.92 (0.86–0.98)	.0085
Nadir CD4 count^b^								
Per 50 cells/mm	0.87 (0.83–0.92)	<.0001	0.89 (0.84–0.94)	<.0001	0.87 (0.79–0.96)	.0066	0.85 (0.76–0.96)	.007
AIDS^b^								
No	1				1			
Yes	1.20 (0.90–1.61)	.2175			1.51 (0.89–2.55)	.1278		
ART/viral load^b^								
ART-naive/VL >10000	1		1		1		1	
ART-naive/VL <10000	0.24 (0.13–0.46)	<.0001	0.28 (0.14–0.58)	.0005	0.32 (0.10–1.01)	.0512	0.53 (0.16–1.73)	.2919
ART-experienced/VL <50	0.22 (0.15–0.32)	<.0001	0.18 (0.11–0.28)	<.0001	0.22 (0.11–0.46)	<.0001	0.34 (0.14–0.82)	.0162
ART-experienced/VL 51–1000	0.44 (0.27–0.73)	.0014	0.29 (0.16–0.52)	<.0001	0.33 (0.11–0.96)	.0426	0.41 (0.13–1.28)	.1233
ART-experienced/VL 1000–10000	0.42 (0.22–0.82)	.0111	0.35 (0.17–0.71)	.0037	0.59 (0.19–1.89)	.3743	0.72 (0.22–2.41)	.5942
ART-experienced/VL >10000	0.66 (0.40–1.08)	.0988	0.40 (0.22–0.70)	.0015	1.11 (0.47–2.62)	.8095	1.02 (0.40–2.59)	.9725
Current TDF^b^								
No	1		1		1		1	
Yes	0.35 (0.25–0.48)	<.0001	0.41 (0.29–0.58)	<.0001	0.06 (0.02–0.2)	<.0001	0.05 (0.01–0.21)	<.0001
Current PI^b^								
No	1		1		1			
Yes	1.11 (0.85–1.46)	.4451	1.50 (1.08–2.08)	.0153	1.01 (0.6–1.7)	.974		

Abbreviations: AIDS, acquired immunodeficiency syndrome; ART, antiretroviral therapy; CI, confidence interval; CKD, chronic kidney disease; ESKD, end-stage kidney disease; HIV, human immunodeficiency virus; IRR, incidence rate ratio; PI, protease inhibitor; TDF, tenofovir disoproxil fumarate; VL, viral load.

^a^ Poisson regression analysis; multivariable models included region, age, sex, mode of HIV acquisition, current and nadir CD4 cell count, immunovirological status, and TDF exposure (CKD only). For all parameters except age and CD4 count, the IRR is reported in relation to the first parameter in each group.

^b^Time updated variable.

## DISCUSSION

This is, to our knowledge, the first study to directly compare the rate of kidney disease progression in different African populations. After adjusting for demographic and HIV-specific parameters, we observed marked regional differences among people from sub-Saharan Africa who were resident in Britain, with prevalent CKD, incident CKD, and ESKD 2.7 to 6.4-fold more common among West Africans compared to East Africans, and intermediate rates of CKD and ESKD in Southern Africans. The shared ancestry and similar rates of CKD and ESKD among West Africans and Caribbeans suggest that genetic factors are likely to be a major determinant of CKD, and particularly ESKD, in the setting of HIV.

Studies in African Americans have found *APOL1* risk alleles to be associated with focal and segmental glomerulosclerosis (FSGS) and hypertension-attributed ESKD, proteinuria, impaired renal function, and kidney disease progression, and with more-severe histological abnormalities (glomerulosclerosis, interstitial fibrosis, and tubular atrophy) in those with FSGS or proteinuria [9]. *APOL1* gene expression is regulated by inflammatory cytokines that are abundantly present in people with uncontrolled HIV viraemia, immunodeficiency, and (opportunistic) infections, the classical setting in which HIVAN is diagnosed in the UK [4]. CD4 T-lymphocytopenia and HIV replication were independent risk factors for CKD and ESKD in our study participants, suggesting that HIVAN may have accounted for a substantial number of CKD/ESKD cases.

Contemporary cohorts have identified age, diabetes mellitus, hypertension, hepatitis B and C coinfection, and exposure to potentially nephrotoxic medications (especially TDF, ritonavir-boosted atazanavir, and ritonavir-boosted lopinavir) as risk factors for CKD and/or kidney disease progression in (predominantly white) people with HIV [9]. In our African and Caribbean participants, we confirmed the association of CKD with older age, which in part reflects age-related CKD risk factors such as diabetes and cardiovascular disease for which no data were available. Although TDF exposure has been associated with CKD in numerous cohorts including white UK CHIC participants (data not shown), we observed no such association in African and Caribbean participants. A low or lesser susceptibility to the nephrotoxic effects of TDF in sub-Saharan African populations is consistent with data from the Development of Antiretroviral Therapy (DART) trial [11], cohort data from Zambia [12], and a recent study of TDF-associated tubulopathy in which black ethnicity was associated with an 81% reduced risk [13]. The strong negative association between TDF exposure and severe CKD/ESKD, however, may be a reflection of clinical practice in which TDF is discontinued in people whose eGFR approaches (or has fallen to just below) 60 mL/min/1.73 m^2^ [14].

Our study has several limitations. Firstly, the UK CHIC study does not contain information on hypertension, diabetes, cardiovascular disease, renal diagnoses, or concomitant medications. No biological sample collection was available to confirm a contribution of regional differences in *APOL1* polymorphisms or other genetic variability. Although eGFR estimates using CKD-EPI are generally preferred, these have not been well validated in African populations; they are also affected by drugs that inhibit creatinine secretion although this is unlikely to have impacted the results for ESKD. We had to exclude 60% of black people in the UK CHIC cohort because of insufficient information on country of birth or renal function, and, as this was an observational cohort study, we are unable to exclude that bias or confounding may have affected our results. Our findings should thus be regarded as hypothesis generating and require confirmation in African and Caribbean settings. However, as all our participants were aware of their HIV status and had unrestricted access to ART and dialysis, the rates of CKD and ESKD may be even higher in sub-Saharan Africa and the Caribbean.

In conclusion, consistent with a recent meta-analysis of 61 studies in HIV-positive populations [15], we observed notably higher rates of CKD in West Africans and Caribbeans, 2 populations with shared ancestry, suggesting that genetic factors are likely to be important CKD risk factors in the setting of HIV. As immunovirological control was an important additional CKD risk factor and levels of HIV diagnosis, ART coverage, and viral suppression, especially in West Africa and the Caribbean, remain well below the 90-90-90 target set by UNAIDS, the scale up of HIV diagnosis, treatment, and prevention programs in these regions should be prioritized.

## Supplementary Data

Supplementary materials are available at *The Journal of Infectious Diseases* online. Consisting of data provided by the authors to benefit the reader, the posted materials are not copyedited and are the sole responsibility of the authors, so questions or comments should be addressed to the corresponding author.

Supplementary Material

## Notes

Presented in part: Conference on Retroviruses and Opportunistic Infections (CROI) 2018, 4–7 March 2018, Boston, poster 731.

## Notes

***Author contributions.*** F. A. P. designed the study. S. J. undertook the statistical analysis with input from A. N. P., C. A. S., and F. A. P. The first draft of the manuscript was written by F. A. P. All authors contributed to and approved the final version of the manuscript.

***Acknowledgments.*** Members of the UK Collaborative HIV Cohort (CHIC) Steering Committee: Jonathan Ainsworth, Sris Allan, Jane Anderson, Abdel Babiker, David Chadwick, Duncan Churchill, Valerie Delpech, David Dunn, Brian Gazzard, Richard Gilson, Mark Gompels, Phillip Hay, Teresa Hill, Margaret Johnson, Sophie Jose, Stephen Kegg, Clifford Leen, Fabiola Martin, Dushyant Mital, Mark Nelson, Chloe Orkin, Adrian Palfreeman, Andrew Phillips, Deenan Pillay, Frank Post, Jillian Pritchard, Caroline Sabin, Achim Schwenk, Anjum Tariq, Roy Trevelion, Andy Ustianowski, John Walsh. Additional UK CHIC investigators are listed in the supplementary data.

***Disclaimer.*** The views expressed in this manuscript are those of the researchers and not necessarily those of the MRC.

***Financial support.*** This work, as part of the UK CHIC Study, was supported by the Medical Research Council (MRC) UK (grant numbers G0000199, G0600337, G0900274, and M004236).

***Potential conflicts of interest.*** A. N. P. reports grants from Medical Research Council (MRC) and personal fees from Gilead Sciences. C. A. S. reports grants from MRC and personal fees from Gilead Sciences, ViiV Healthcare, and Janssen-Cilag. R. J. reports grants to Chelsea and Westminster Hospital NHS Foundation Trust from ViiV Healthcare, Merck, and Gilead Sciences, and personal fees from Gilead Sciences, Janssen-Cilag, and ViiV Healthcare. A. W. reports grants to Imperial College London from ViiV Healthcare, Gilead Sciences, Bristol-Myers Squibb, Janssen, and MSD; and speaker fees and advisory boards from ViiV Healthcare, Gilead Sciences, MSD, and Janssen. F. B. reports personal fees from Gilead Sciences. F. A. P. reports grants to King’s College Hospital NHS Foundation Trust from ViiV Healthcare and Gilead Sciences, and personal fees from Gilead Sciences, Janssen-Cilag, GlaxoSmithKline/ViiV Healthcare, and Merck. All other authors report no potential conflicts. All authors have submitted the ICMJE Form for Disclosure of Potential Conflicts of Interest. Conflicts that the editors consider relevant to the content of the manuscript have been disclosed.
