# Supplementary material for: Chronic Kidney Disease Risk in African and Caribbean Populations With HIV
Source: J Infect Dis. 2018 Jul 6;218(11):1767–72. doi: 10.1093/infdis/jiy397 (PMC6195659; doi:10.1093/infdis/jiy397)
Supplement: Supplementary Material [file jiy397_suppl_supplementary_material.docx]

**Supplementary material**

|  |  | **Page** |
| --- | --- | --- |
| **Figure S1** | Disposition of UK CHIC participants for the analyses of CKD in those of black African or Caribbean ethnicity. | **2** |
| **Figure S2** | Kaplan Meier estimates of the (cumulative) probability of CKD and ESKD by region of birth/ethnicity. | **3/4** |
| **Table S1** | Characteristics of Black African population according to region/country of birth | **5/6** |
| **Table S2** | Associations between region of birth/ethnicity group and CKD/ESKD | **7/8** |
| **Appendix** | Appendix: Investigators of the UK CHIC study | **9** |

**Figure S1: Disposition of UK CHIC participants for the analyses of CKD in those of black African or Caribbean ethnicity**

Black ethnicity (n=19,710)

Black African with no country of birth (n=5596)

Black African with country of birth or black Caribbean (n=14,114)

Insufficient creatinine assessments (n=6326)

≥2 eGFR measured >90 days apart (n=7788)

Assessed for baseline characteristics and cumulative incidence CKD ≥3, ≥4 and 5

CKD prior to baseline (n=24)

Assessed for incidence CKD ≥3, ≥4 and 5 (n=7764)

**Figure S2**

Kaplan Meier estimates of the (cumulative) probability of CKD by region of birth/ethnicity. Both prevalent and incident cases are included.


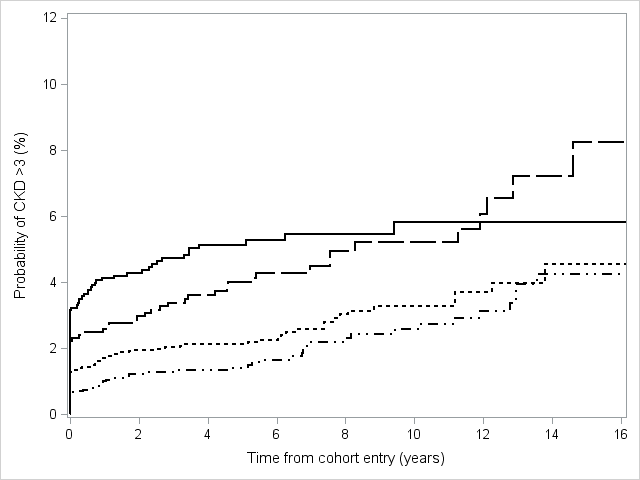


| **Number at risk (CKD ≥3)** | **Baseline** | **2** | **4** | **6** | **8** | **10** | **12** | **14** | **16** |
| --- | --- | --- | --- | --- | --- | --- | --- | --- | --- |
| East | 2033 | 1743 | 1443 | 1100 | 797 | 635 | 458 | 285 | 131 |
| Southern | 3101 | 2537 | 1947 | 1259 | 839 | 568 | 372 | 157 | 52 |
| West | 1487 | 1138 | 833 | 553 | 338 | 213 | 140 | 68 | 32 |
| Caribbean | 1167 | 972 | 792 | 580 | 388 | 297 | 200 | 105 | 60 |
| Total | 7788 | 6390 | 5015 | 3492 | 2362 | 1713 | 1170 | 615 | 275 |

| Ethnicity/region |  | East |  | Southern |  | West |  | Caribbean |
| --- | --- | --- | --- | --- | --- | --- | --- | --- |

**Fig S2 (cont.)**

Kaplan Meier estimates of the (cumulative) probability of ESKD by region of birth/ethnicity. Both prevalent and incident cases are included.


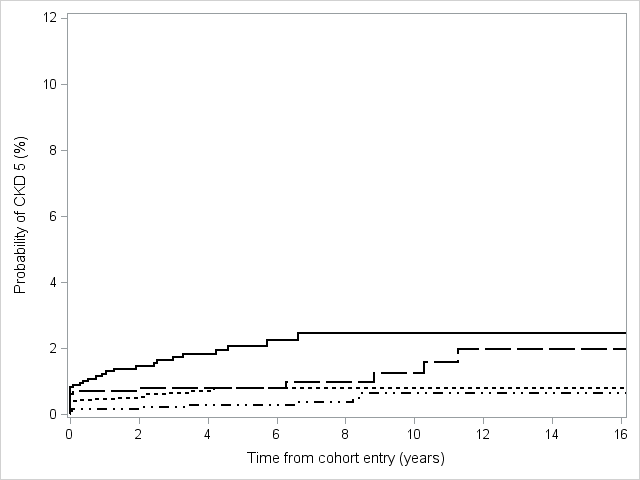


| **Number at risk (CKD 5)** | **Baseline** | **2** | **4** | **6** | **8** | **10** | **12** | **14** | **16** |
| --- | --- | --- | --- | --- | --- | --- | --- | --- | --- |
| East | 2033 | 1764 | 1462 | 1119 | 817 | 648 | 471 | 296 | 137 |
| Southern | 3101 | 2573 | 1974 | 1279 | 858 | 580 | 383 | 163 | 53 |
| West | 1487 | 1170 | 861 | 569 | 348 | 218 | 142 | 69 | 32 |
| Caribbean | 1167 | 992 | 814 | 595 | 399 | 307 | 206 | 110 | 63 |
| Total | 7788 | 6499 | 5111 | 3562 | 2422 | 1753 | 1202 | 638 | 285 |

| Ethnicity/region |  | East |  | Southern |  | West |  | Caribbean |
| --- | --- | --- | --- | --- | --- | --- | --- | --- |

**Table S1: Characteristics of Black African population according to region/country of birth**

|  |  |  |  | Baseline | | | | | |  | Cumulative probability at 5 years^1^ | | | | |
| --- | --- | --- | --- | --- | --- | --- | --- | --- | --- | --- | --- | --- | --- | --- | --- |
|  | **N** | Age | Female | ART | CD4 | eGFR | eGFR <60 |  | eGFR <15 |  | CKD >3 |  | CKD >5 | |  |
|  |  | mean (SD) | N (%) | N (%) | Med (IQR) | Med (IQR) | N (%) |  | N (%) |  | (%) |  | | (%) |  |
| **East Africa** |  |  |  |  |  |  |  |  |  |  |  |  |  | |  |
| Uganda | 1021 | 37 (9.0) | 665 (65.1) | 595 (58.3) | 310 (158, 480) | 112 (93, 129) | 17 (1.7) |  | 1 (0.1) |  | 2.0% |  | 0.4% | |  |
| Kenya | 220 | 36 (8.5) | 151 (68.6) | 103 (46.8) | 310 (170, 472) | 118 (100, 131) | 3 (1.4) |  | 0 |  | 0.5% |  | 0% | |  |
| Ethiopia | 237 | 34 (8.0) | 145 (61.2) | 103 (43.5) | 279 (140, 470) | 131 (119, 142) | 0 |  | 0 |  | 0% |  | 0% | |  |
| Somalia | 166 | 37 (10.4) | 99 (59.6) | 82 (49.4) | 312 (180, 450) | 125 (108, 137) | 2 (1.2) |  | 1 (0.6) |  | 1.7% |  | 0.6% | |  |
| Eritrea (incl. Sudan, n=29) | 134 | 34 (9.2) | 72 (53.7) | 54 (40.3) | 307 (170, 499) | 134 (118, 144) | 0 |  | 0 |  | 0.7% |  | 0% | |  |
| Rwanda | 115 | 33 (8.5) | 78 (67.8) | 60 (52.2) | 304 (190, 449) | 117 (98, 133) | 0 |  | 0 |  | 2.0% |  | 0% | |  |
| Burundi | 80 | 33 (8.1) | 61 (76.3) | 48 (60.0) | 365 (240, 590) | 125 (117, 139) | 0 |  | 0 |  | 0% |  | 0% | |  |
| Tanzania | 60 | 36 (8.1) | 45 (75.0) | 31 (51.7) | 246 (120, 430) | 119 (98, 135) | 1 (1.7) |  | 0 |  | 1.7% |  | 0% | |  |
| **Southern Africa** |  |  |  |  |  |  |  |  |  |  |  |  |  | |  |
| Zimbabwe (incl. Mozambique, n=18) | 1722 | 37 (9.1) | 1191 (69.2) | 900 (52.3) | 334 (180, 500) | 114 (98, 129) | 46 (2.7) |  | 15 (0.9) |  | 2.3% |  | 0.9% | |  |
| South Africa  (incl. Swaziland and Lesotho, n=14) | 299 | 35 (8.2) | 205 (68.6) | 141 (47.2) | 319 (187, 510) | 113 (99, 129) | 4 (1.3) |  | 2 (0.7) |  | 1.7% |  | 0.7% | |  |
| Zambia | 394 | 36 (9.0) | 277 (70.3) | 204 (51.8) | 271 (140, 467) | 110 (93, 127) | 12 (3.1) |  | 0 |  | 2.4% |  | 0.7% | |  |
| Congo  (DRC and Rep of Congo, n=268/9) | 277 | 36 (9.5) | 177 (63.9) | 168 (60.7) | 290 (177, 458) | 119 (97, 132) | 4 (1.4) |  | 1 (0.4) |  | 2.0% |  | 1.4% | |  |
| Malawi | 153 | 35 (9.3) | 102 (66.7) | 66 (43.1) | 275 (157, 487) | 119 (100, 136) | 5 (3.3) |  | 0 |  | 1.4% |  | 0% | |  |
| Cameroon | 125 | 33 (7.8) | 80 (64.0) | 48 (38.4) | 329 (184, 450) | 115 (96, 1.32) | 3 (2.4) |  | 2 (1.6) |  | 0.8% |  | 0.8% | |  |
| Angola | 78 | 32 (8.8) | 42 (53.9) | 39 (50.0) | 335 (182, 500) | 129 (110, 338) | 0 |  | 0 |  | 0% |  | 0% | |  |
| Botswana (incl. Namibia, n=9) | 53 | 35 (6.9) | 41 (77.4) | 29 (54.7) | 293 (169, 429) | 123 (104, 132) | 1 (1.9) |  | 1 (1.9) |  | 1.9% |  | 0% | |  |
| **West Africa** |  |  |  |  |  |  |  |  |  |  |  |  |  | |  |
| Nigeria | 721 | 36 (9.6) | 425 (59.0) | 265 (36.8) | 285 (138, 448) | 112 (90, 131) | 37 (5.1) |  | 12 (1.7) |  | 6.3% |  | 3.0% | |  |
| Ghana (incl. Togo and Benin, n=16) | 368 | 39 (10.3) | 212 (57.6) | 171 (46.5) | 336 (171, 486) | 110 (94, 129) | 22 (6.0) |  | 4 (1.1) |  | 5.1% |  | 1.1% | |  |
| Cote D'Ivoire | 194 | 33 (7.7) | 123 (63.4) | 70 (36.1) | 284 (115, 450) | 116 (99, 131) | 5 (2.6) |  | 1 (0.5) |  | 2.3% |  | 1.7% | |  |
| Sierra Leone  (incl. Guinea, Liberia, Senegal and Gambia, n=67) | 204 | 35 (9.9) | 109 (53.4) | 59 (28.9) | 304 (145, 475) | 114 (99, 130) | 11 (5.4) |  | 1 (0.5) |  | 3.4% |  | 1.0% | |  |

^1^Kaplan-Meier estimates (includes baseline cases), eGFR = estimated glomerular filtration rate, CKD = chronic kidney disease, CKD >3, >4 and 5 was defined by an eGFR <60 mL/min/1.73m^2^ for >3 months

**Table S2 Associations between region of birth/ethnicity group and CKD/ESKD with adjustment for HIV subtype**

|  |  |  |  |  |  |  |  |  |  |
| --- | --- | --- | --- | --- | --- | --- | --- | --- | --- |
|  | **CKD (eGFR <60 mL/min/1.73m^2^ threshold)** | | | |  | **ESKD (eGFR <15 mL/min/1.73m^2^ threshold)** | | | |
|  | *Univariable estimates^1^* | | *Multivariable estimates^1^* | |  | *Univariable estimates^1^* | | *Multivariable estimates^1^* | |
|  | *IRR (95% CI)* | *P-value* | *IRR (95% CI)* | *P-value* |  | *IRR (95% CI)* | *P-value* | *IRR (95% CI)* | *P-value* |
| **Region** | | | | |  |  | | | |
| East Africa | 1 |  | 1 |  |  | 1 |  | 1 |  |
| Southern Africa | 1.55 (1.06, 2.27) | 0.0229 | 1.35 (0.78, 2.35) | 0.2838 |  | 2.60 (1.11, 6.08) | 0.0278 | 2.75 (0.82, 9.19) | 0.1013 |
| West Africa | 2.95 (1.99, 4.36) | <.0001 | 1.74 (0.95, 3.20) | 0.0748 |  | 6.14 (2.63, 14.3) | <.0001 | 6.04 (1.68, 21.70) | 0.0059 |
| Caribbean | 2.30 (1.52, 3.48) | <.0001 | 1.46 (0.74, 2.89) | 0.2753 |  | 3.55 (1.42, 8.89) | 0.0069 | 6.58 (1.71, 25.37) | 0.0062 |
| **Age*** | | | | |  |  | | | |
| per 10 years | 1.83 (1.62, 2.07) | <.0001 | 2.20 (1.90, 2.57) | <.0001 |  | 1.11 (0.86, 1.43) | 0.4395 | 1.26 (0.91, 1.74) | 0.1694 |
| **Sex** | | | | |  |  | | | |
| Male | 1 |  | 1 |  |  | 1 |  | 1 |  |
| Female | 0.74 (0.57, 0.96) | 0.0238 | 1.05 (0.73, 1.50) | 0.7976 |  | 0.57 (0.35, 0.93) | 0.024 | 0.62 (0.32, 1.19) | 0.1529 |
| **Mode of HIV Acquisition** | | | | |  |  | | | |
| Heterosexual | 1 |  |  |  |  | 1 |  | 1 |  |
| Other | 0.54 (0.34, 0.86) | 0.0088 |  |  |  | 0.38 (0.14, 1.04) | 0.0596 | 0.20 (0.04, 0.94) | 0.0416 |
| **Current CD4 count*** | | | | |  |  | | | |
| per 50 cells/mm | 0.86 (0.84, 0.89) | <.0001 | 0.93 (0.89, 0.97) | 0.0002 |  | 0.85 (0.8, 0.90) | <.0001 | 0.92 (0.85, 1.00) | 0.0388 |
| **Nadir CD4 count*** | | | | |  |  | | | |
| per 50 cells/mm | 0.87 (0.83, 0.92) | <.0001 | 0.89 (0.82, 0.96) | 0.0017 |  | 0.87 (0.79, 0.96) | 0.0066 | 0.92 (0.81, 1.05) | 0.2315 |
| **AIDS*** | | | | |  |  | | | |
| No | 1 |  |  |  |  | 1 |  |  |  |
| Yes | 1.20 (0.90, 1.61) | 0.2175 |  |  |  | 1.51 (0.89, 2.55) | 0.1278 |  |  |
| **ART/Viral load*** | | | | |  |  | | | |
| ART-naive/VL >10,000 | 1 |  | 1 |  |  | 1 |  | 1 |  |
| ART-naive/VL <10,000 | 0.24 (0.13, 0.46) | <.0001 | 0.40 (0.18, 0.86) | 0.0189 |  | 0.32 (0.10, 1.01) | 0.0512 | 0.50 (0.13, 1.97) | 0.3238 |
| ART-experienced/VL <50 | 0.22 (0.15, 0.32) | <.0001 | 0.20 (0.11, 0.35) | <.0001 |  | 0.22 (0.11, 0.46) | <.0001 | 0.16 (0.06, 0.47) | 0.0008 |
| ART-experienced/VL 51-1,000 | 0.44 (0.27, 0.73) | 0.0014 | 0.37 (0.19, 0.72) | 0.0035 |  | 0.33 (0.11, 0.96) | 0.0426 | 0.27 (0.07, 0.99) | 0.0481 |
| ART-experienced/VL 1,000-10,000 | 0.42 (0.22, 0.82) | 0.0111 | 0.48 (0.22, 1.04) | 0.0619 |  | 0.59 (0.19, 1.89) | 0.3743 | 0.65 (0.18, 2.37) | 0.5163 |
| ART-experienced/VL >10,000 | 0.66 (0.40, 1.08) | 0.0988 | 0.49 (0.25, 0.93) | 0.0301 |  | 1.11 (0.47, 2.62) | 0.8095 | 1.04 (0.37, 2.94) | 0.9389 |
| **Current TDF*** | | | | |  |  | | | |
| No | 1 |  | 1 |  |  | 1 |  |  |  |
| Yes | 0.35 (0.25, 0.48) | <.0001 | 0.44 (0.29, 0.67) | 0.0001 |  | 0.06 (0.02, 0.2) | <.0001 |  |  |
| **Current PI*** | | | | |  |  | | | |
| No | 1 |  |  |  |  | 1 |  |  |  |
| Yes | 1.11 (0.85, 1.46) | 0.4451 |  |  |  | 1.01 (0.6, 1.7) | 0.974 |  |  |
| **Subtype** | | | | |  |  | | | |
| A | 1 |  | 1 |  |  | 1 |  | 1 |  |
| B | 1.98 (0.98, 3.97) | 0.0555 | 1.81 (0.78, 4.22) | 0.1684 |  | 1.71 (0.41, 7.17) | 0.461 | 0.89 (0.17, 4.58) | 0.8847 |
| C | 2.00 (1.07, 3.73) | 0.0291 | 2.06 (1.00, 4.24) | 0.051 |  | 2.51 (0.74, 8.52) | 0.1398 | 1.94 (0.51, 7.35) | 0.3284 |
| CRF | 3.00 (1.57, 5.74) | 0.0009 | 2.48 (1.16, 5.30) | 0.0191 |  | 4.06 (1.16, 14.26) | 0.0286 | 1.46 (0.35, 6.12) | 0.6031 |
| Other | 2.63 (1.31, 5.25) | 0.0063 | 2.41 (1.14, 5.11) | 0.0218 |  | 3.01 (0.78, 11.66) | 0.1098 | 1.60 (0.37, 6.92) | 0.5308 |

*Time updated variable

^1^ Poisson regression analysis (multivariable models included region, age, sex, mode of HIV acquisition (ESKD only), current and nadir CD4 cell count, immunovirological status, TDF exposure (CKD only), and HIV subtype). For all parameters except age and CD4 count, the IRR is reported in relation to the first parameter in each group.

IRR=Incidence rate ratio, CKD = chronic kidney disease, ESKD = end-stage kidney disease, ART = antiretroviral therapy, VL = viral load, TDF = tenofovir disoproxil fumarate, PI = protease inhibitor, CRF = circulating recombinant form

***Appendix: Investigators of the UK CHIC study***

***Steering Committee:*** Jonathan Ainsworth, Sris Allan, Jane Anderson, Abdel Babiker, David Chadwick, Duncan Churchill, Valerie Delpech, David Dunn, Brian Gazzard, Richard Gilson, Mark Gompels, Phillip Hay, Teresa Hill, Margaret Johnson, Sophie Jose, Stephen Kegg, Clifford Leen, Fabiola Martin, Dushyant Mital, Mark Nelson, Chloe Orkin, Adrian Palfreeman, Andrew Phillips, Deenan Pillay, Frank Post, Jillian Pritchard, Caroline Sabin, Achim Schwenk, Anjum Tariq, Roy Trevelion, Andy Ustianowski, John Walsh.

***Central Co-ordination****: University College London* (Teresa Hill, Sophie Jose, Andrew Phillips, Caroline Sabin, Alicia Thornton, Susie Huntington); *Medical Research Council Clinical Trials Unit at UCL, London* (David Dunn, Adam Glabay, Shaadi Shidfar).

***Participating Centres:*** *Barts Health NHS Trust, London* (Chloe Orkin, Janet Lynch, James Hand, Carl de Souza); *Brighton and Sussex University Hospitals NHS Trust*, Brighton* (Duncan Churchill, Nicky Perry, Stuart Tilbury, Elaney Youssef); *Chelsea and Westminster Hospital NHS Foundation Trust*, London* (Mark Nelson, Tracey Mabika, David Asboe, Sundhiya Mandalia); *Homerton University Hospital NHS Trust, London* (Jane Anderson, Sajid Munshi); *King’s College Hospital NHS Foundation Trust*, London* (Frank Post, Ade Adefisan, Chris Taylor, Zachary Gleisner, Fowzia Ibrahim, Lucy Campbell); Middlesbrough, South Tees Hospitals NHS Foundation Trust, (David Chadwick, Kirsty Baillie); *Mortimer Market Centre, Central and North West London NHS Foundation Trust/University College London*, London* (Richard Gilson, Nataliya Brima, Ian Williams); *North Middlesex University Hospital NHS Trust*, London* (Jonathan Ainsworth, Achim Schwenk, Sheila Miller, Chris Wood); *Royal Free NHS Foundation Trust/University College London*, London* (Margaret Johnson, Mike Youle, Fiona Lampe, Colette Smith, Rob Tsintas, Clinton Chaloner, Samantha Hutchinson, Caroline Sabin, Andrew Phillips, Teresa Hill, Sophie Jose); *Imperial College Healthcare NHS Trust*, London* (John Walsh, Nicky Mackie, Alan Winston, Jonathan Weber, Farhan Ramzan, Mark Carder); *The Lothian University Hospitals NHS Trust*, Edinburgh* (Clifford Leen, Alan Wilson, Sheila Morris); *North Bristol NHS Trust* (Mark Gompels, Sue Allan); *Leicester,* *University Hospitals of Leicester NHS Trust* (Adrian Palfreeman, Adam Lewszuk); *Woolwich,* *Lewisham and Greenwich NHS Trust* (Stephen Kegg, Akin Faleye, Victoria Ogunbiyi, Sue Mitchell); *St. George’s Healthcare NHS Trust*, London* (Phillip Hay, Christian Kemble); *York Teaching Hospital NHS Foundation Trust*, York* (Fabiola Martin, Sarah Russell-Sharpe, Janet Gravely); *University Hospitals Coventry and Warwickshire NHS Trust*, Coventry* (Sris Allan, Andrew Harte); *The Royal Wolverhampton Hospitals NHS Trust*, Wolverhampton* (Anjum Tariq, Hazel Spencer, Ron Jones); *Ashford and St.Peter’s Hospitals NHS Foundation Trust*, Chertsey* (Jillian Pritchard, Shirley Cumming, Claire Atkinson); *Milton Keynes Hospital NHS Foundation Trust*, Milton Keynes* (Dushyant Mital, Veronica Edgell, Julie Allen); *The Pennine Acute Hospitals NHS Trust* (Andy Ustianowski, Cynthia Murphy, Ilise Gunder); *Public Health England, London* (Valerie Delpech); *i-Base* (Roy Trevelion).

*These centres contributed data to the presented analyses
